# Supplementary material for: Transgenic mice expressing tunable levels of DUX4 develop characteristic facioscapulohumeral muscular dystrophy-like pathophysiology ranging in severity
Source: Skelet Muscle. 2020 Apr 11;10:8. doi: 10.1186/s13395-020-00227-4 (PMC7149937; doi:10.1186/s13395-020-00227-4)
Supplement: Supplementary file 1 — Additional file 1: Figure S1.FLExDUX4 transgenic mice kept in different housing facilities acquire different mouth and gut microbiomes and have differing alopecia and GI health. Figure S2. Mosaic tamoxifen dose-dependent recombination in gastrocnemius muscle of ACTA1-MCM;R26NZG bi-transgenic mice. Figure S3. Increased TMX dosage leads to increased mosaic recombination in skeletal muscle of ACTA1-MCM;R26NZG bi-transgenic mice. Figure S4. There is no significant difference in the transgene recombination rate between male and female ACTA1-MCM/FLExD bi-transgenic FSHD-like mice. Figure S5. Quantification of DUX4-FL protein positive myonuclei. Figure S6. The moderate and severe FSHD-like mouse models show significant weight loss. Figure S7. Maximum isometric forces of the female FSHD-like mouse models. Figure S8. Maximum and specific isometric forces of the male mild and severe FSHD-like mouse models. Figure S9. Pax7 target genes are not significantly misexpressed in the FSHD-like mouse models. Figure S10. GO enrichment analysis of differentially expressed genes (>2-fold) in the different FSHD-like severity model mice. Figure S11. Fiber number per cross-section does not significantly change with severity. Figure S12. The heart is not affected by TMX treatment in control or bi-transgenic animals. Figure S13. Quantification of eMyHC positive muscle cells in different FSHD-like mouse models. Figure S14.Mstn gene mRNA expression decreases with increased DUX4-fl expression in the FSHD-like mouse models. Figure S15. Quantification of TUNEL positive nuclei in FSHD-like mouse models. Figure S16. Quantification of SR staining shows significant fibrosis in late stages of the moderate and severe FSHD-like mouse models. Figure S17. Cardiac muscle from FSHD-like mouse models shows no signs of increased fibrosis. [file 13395_2020_227_MOESM1_ESM.pdf]

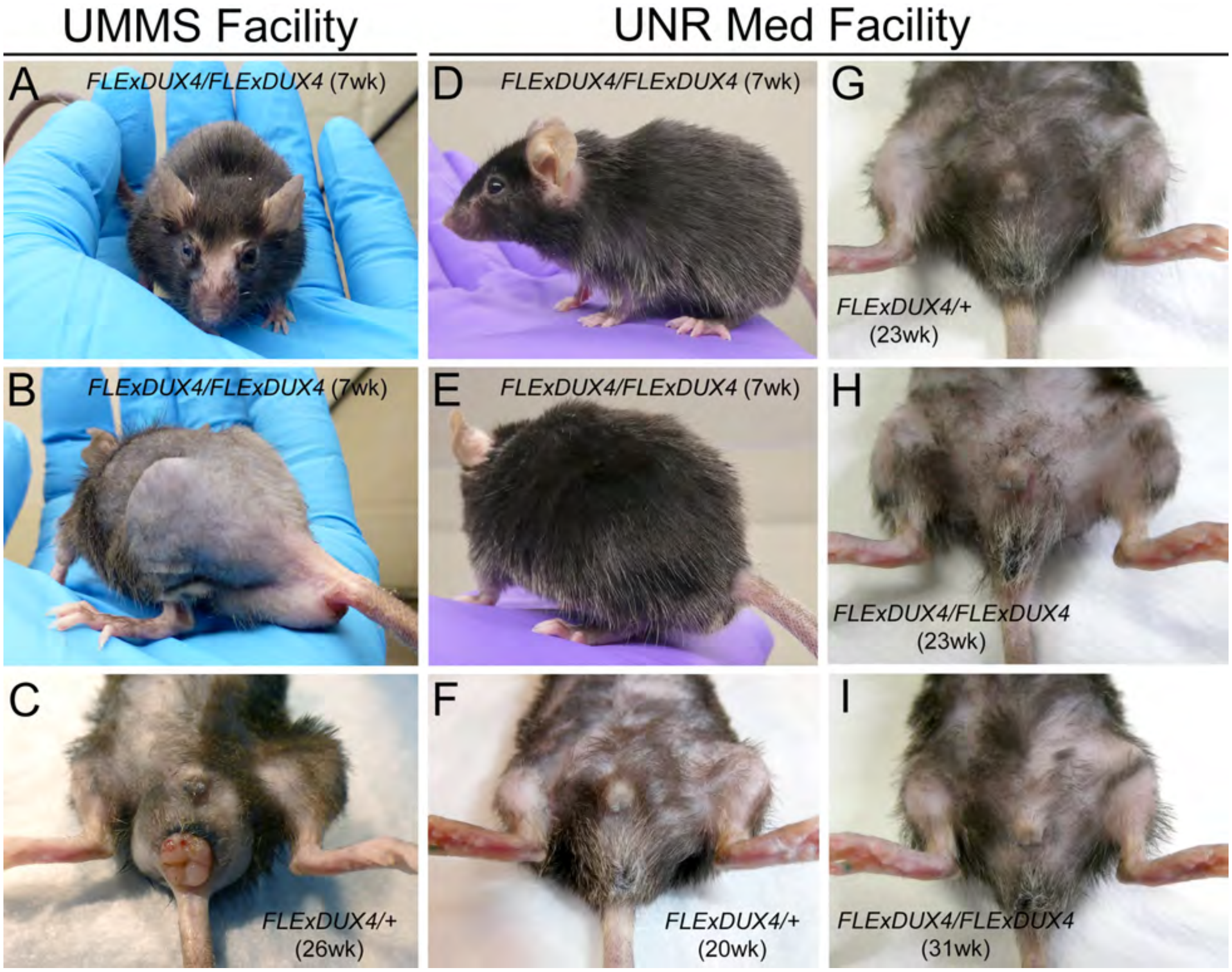

Figure S1: ***FLExDUX4*** **transgenic mice kept in different housing facilities acquire different mouth and gut microbiomes and have differing alopecia and GI health. A, B)** Male homozygous *FLExD/FLExD* mice at 7 weeks and **C)** hemizygous *FLExD*/+ mice at 26 weeks of age housed in a UMMS A-Level mouse facility have severe alopecia and rectal prolapse. **D, E, H, I)** Male homozygous *FLExD/FLExD* mice, 7, 7, 23, and 31 weeks respectively, and **F, G)** hemizygous *FLExD*/+ mice, 20 and 23 weeks old respectively, housed exclusively at the UNR CMM facility have very mild alopecia and no rectal prolapse are found.

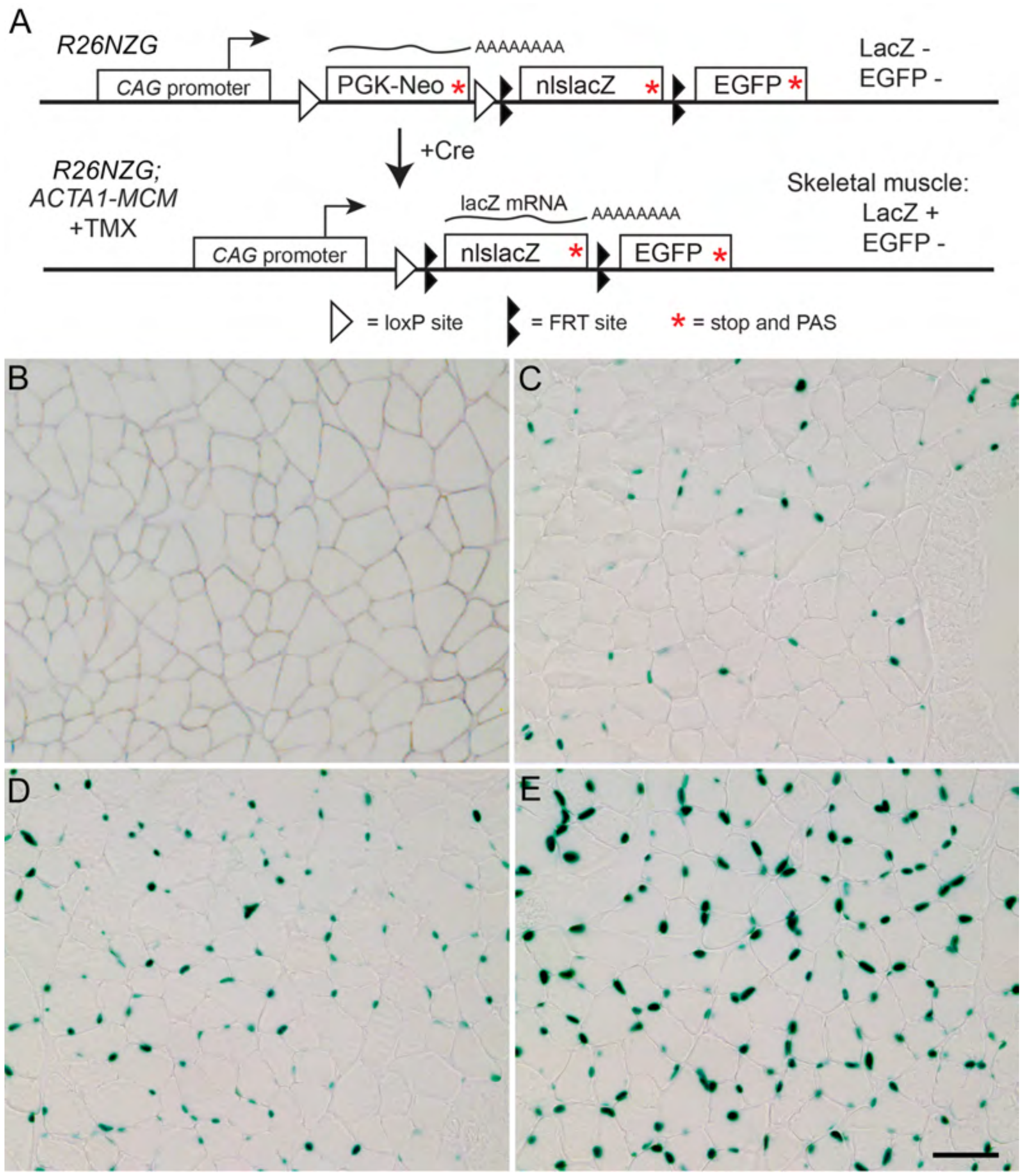

Figure S2: Mosaic tamoxifen dose-dependent recombination in gastrocnemius muscle of *ACTA1-MCM;R26^{NZG}* bi-transgenic mice. A) Cartoon depicting the cre-induced nuclear ß-galactosidase (nLacZ) expression from the *R26^{NZG}* reporter mice. *ACTA1-MCM* mice express MerCreMer, a tamoxifen (TMX)-inducible Cre DNA recombinase under control of the skeletal muscle specific human skeletal actin gene (*ACTA1*) promoter. In *ACTA1-MCM; R26^{NZG}* bi-transgenic mice, MerCreMer protein translocate to nucleus upon TMX binding and the resulting recombination causes the deletion of the loxP-flanked PGK-Neo cassette and expression of a nuclear localized ß-galactosidase protein, which is detected by X-Gal (blue) staining. B-E) X-Gal staining of cross sections of gastrocnemius muscles. B) The negative control *R26^{NZG}*/+ mice injected with tamoxifen. C) *ACTA1-MCM; R26^{NZG}* mice have a low mosaic pattern of X-Gal signal in the absence of TMX induction indicating a very low percentage of cells have leaky MerCreMer protein translocation to the nucleus. *ACTA1-MCM; R26^{NZG}* mice with one intraperitoneal (IP) injection of 5 mg/kg TMX (D) or two IP injections of 10 mg/kg TMX (E) show dose-dependent increases in recombined nuclei expressing nLacZ. All sections were stained with X-Gal for 50 minutes. Scale bar = 50 μm.

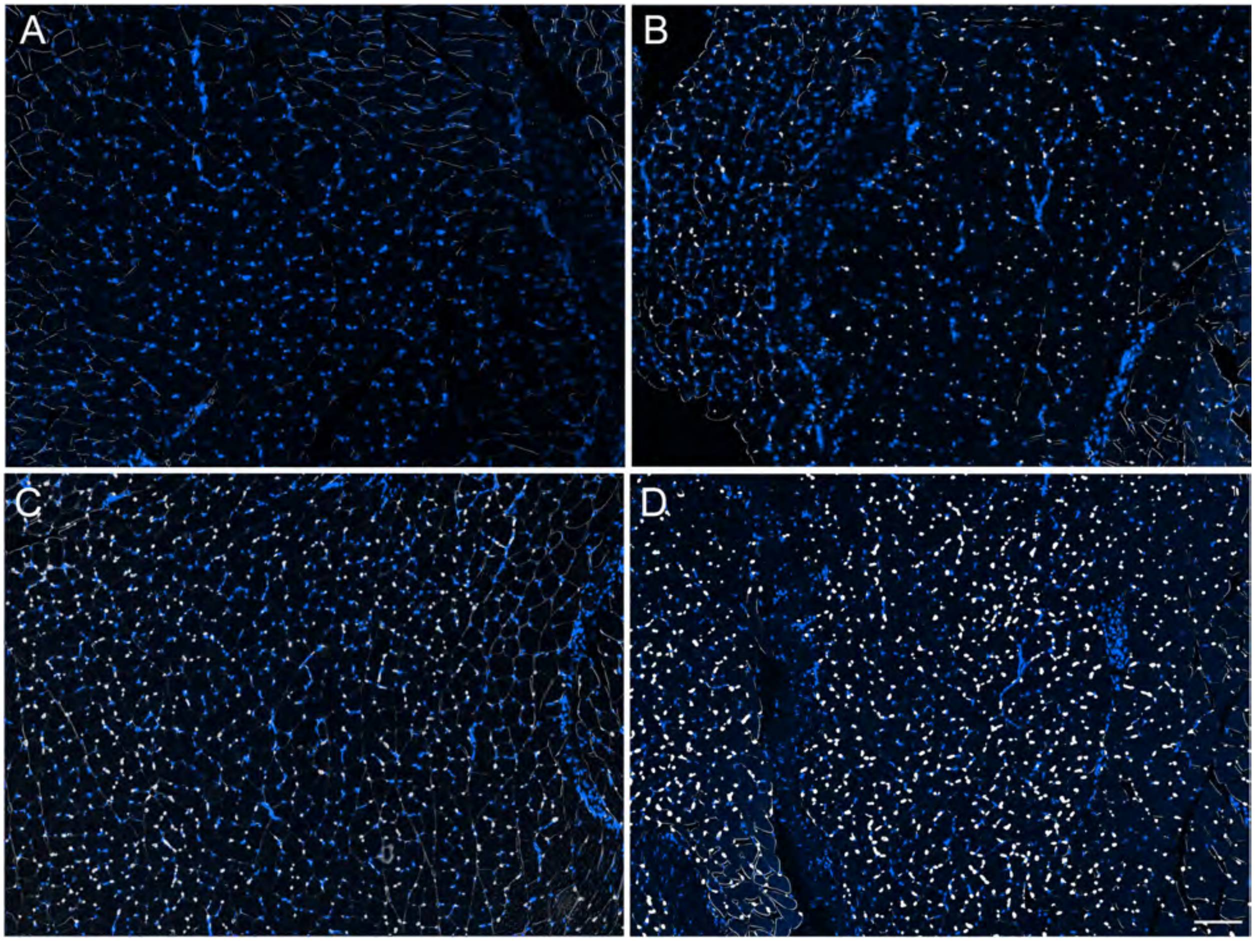

Figure S3: Increased TMX dosage leads to increased mosaic recombination in skeletal muscle of *ACTA1-MCM;R26^{NZG}* bi-transgenic mice. Inverted images of X-Gal signal (white) overlaid with the DAPI signal (blue) were used to identify the nuclear recombination frequency in gastrocnemius muscle sections, described in Figure S1. A) The negative control *ACTA1-MCM* mice have no LacZ expression. B) Bi-transgenic *ACTA1-MCM; R26^{NZG}* mice in the absence of TMX show a low mosaic pattern of nuclear LacZ expression (231.5 X-Gal positive nuclei/mm^2). C) Low level and D) high dose of TMX show 668.5 and 941.9 X-Gal positive nuclei/mm^2, respectively. Scale bar = 100 μm

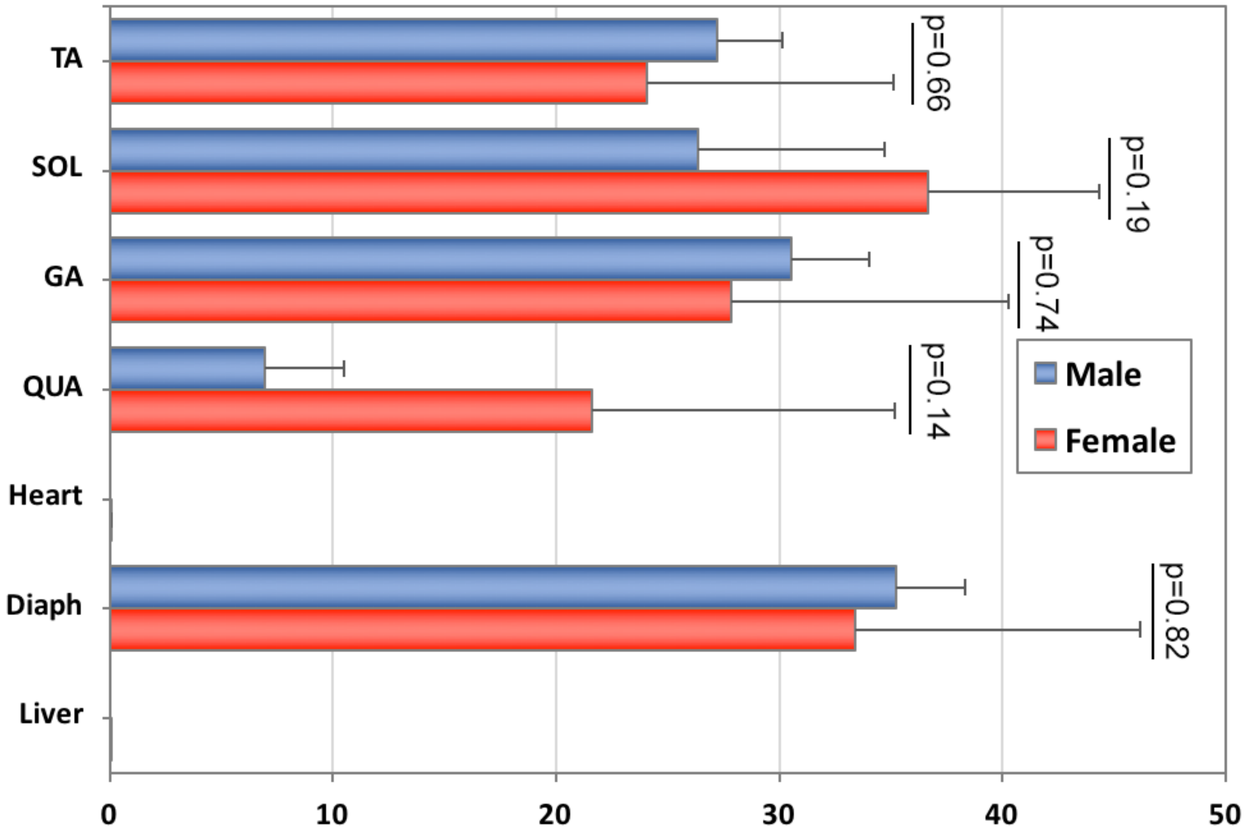

Figure S4: There are no significant differences in the transgene recombination rates between male and female *ACTA1-MCM/FLExD* bi-transgenic mice. Transgene recombination was assessed as described in the methods for 8 skeletal muscles, heart and liver for both male (n=3) and female (n=3) mice in the moderate FSHD-like model. Data is mean +/- S.D.

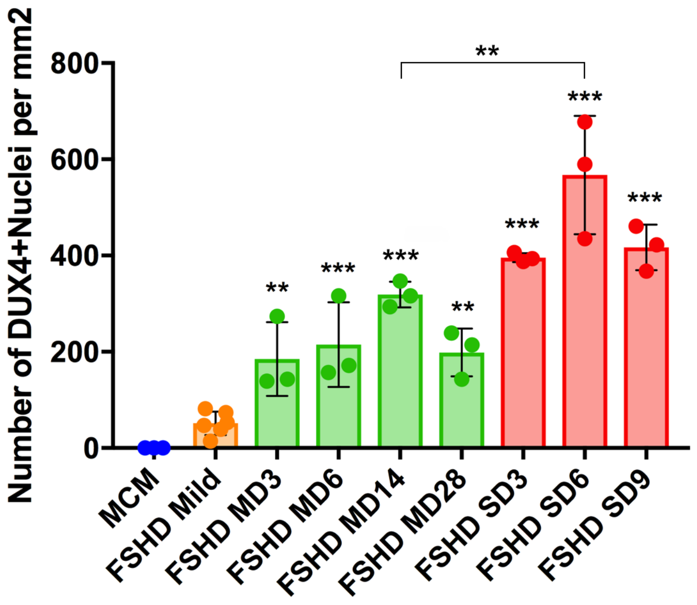

Figure S5: Quantification of DUX4-FL protein positive myonuclei. TA muscle cryosections were immunostained with anti-DUX4 antibody and the number of positively stained myonuclei per mm2 was determined. Data are mean +/- S.D. with significance compared to the mild model using one-way ANOVA, uncorrected Fisher's LSD; $p<0.01$ (**), $p<0.001$ (***).

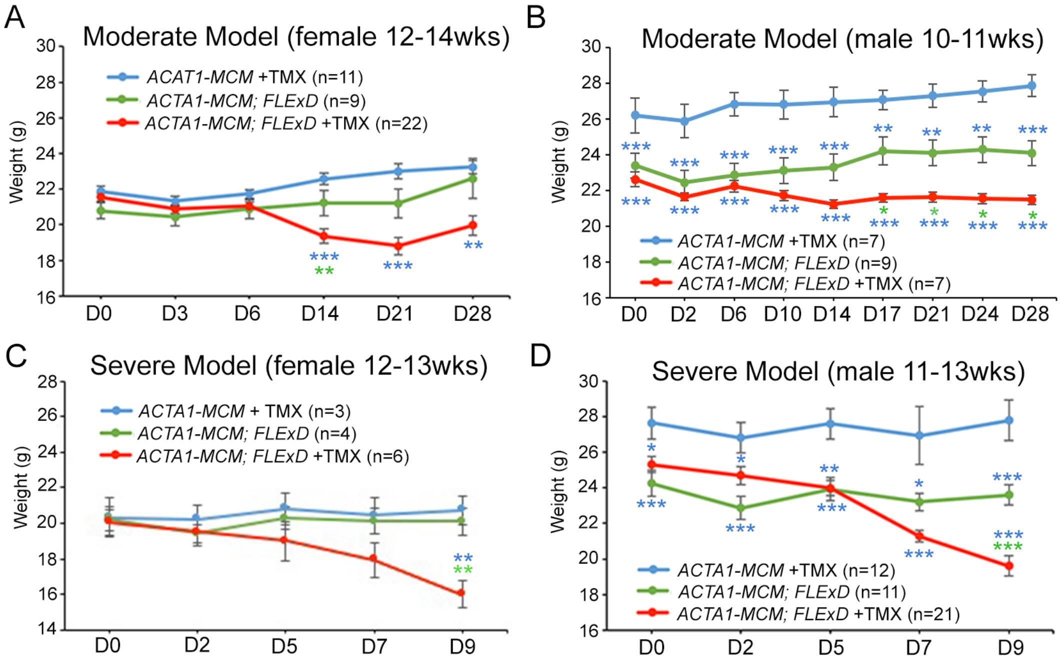

Figure S6: The moderate and severe FSHD-like mouse models show significant weight loss. Mice were weighed during time courses of TMX treatment prior to treadmill running (Figure 4). A) The female moderate model mice (red line) were assayed prior to TMX injection (D0) and 3, 6, 10, 16, 23 and 29 days post-injection (DPI) and compared with age/sex-matched mild model mice (green line) and *ACTA1-MCM* mice injected with TMX (blue line). B) The male moderate model mice (red line) were assayed prior to TMX injection (D0) and 2, 6, 10, 14, 17, 21, 24 and 28 DPI and compared with age/sex-matched mild model mice (green line) and *ACTA1-MCM* mice injected with TMX (blue line). C) The female severe model mice (red line) were assayed prior to TMX injection (D0) and 2, 5, 7, and 8 DPI and compared with age/sex-matched mild model mice (green line) and *ACTA1-MCM* mice injected with TMX (blue line). D) The male severe model mice (red line) were assayed prior to TMX injection (D0) and 2, 5, 7, and 8 DPI and compared with age/sex-matched mild model mice (green line) and *ACTA1-MCM* mice injected with TMX (blue line). The numbers of mice (n) for each experiment are same as in Figure 4. Data is mean +/- S.D. with significance calculated between bi-transgenic + TMX and bi-transgenic no TMX (green) or *ACTA1-MCM* + TMX (blue); * = p<0.05, ** = p<0.01, *** = p<.001.

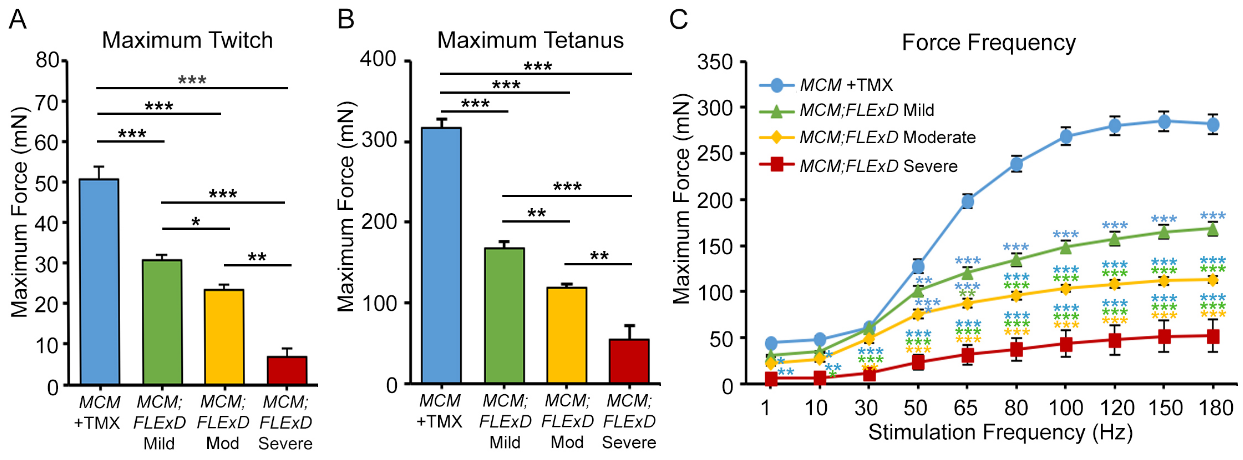

Figure S7: Maximum isometric forces of the female FSHD-like mouse models. EDL muscles from *ACTA1-MCM* +TMX (*MCM* +TMX, control, blue, n=9), mild model (*MCM;FLExD* mild, green, n=9), moderate model (*MCM;FLExD* Mod, yellow, n=6), and severe model mice (*MCM;FLExD* Severe, red, n=3) were assayed at MD14 or SD10 for A) maximum twitch, B) maximum tetanus, C) force frequency, and then normalized to muscle CSA to provide specific force measurements (Figure 5A-C). Data is mean +/- S.D. Significance = * p<0.05, ** p<0.01, ***p<.001; blue asterisks indicate significance compared with *MCM* +TMX control, green asterisks indicate significance when compared with mild model, and yellow asterisks indicate significance when compared with moderate model mice.

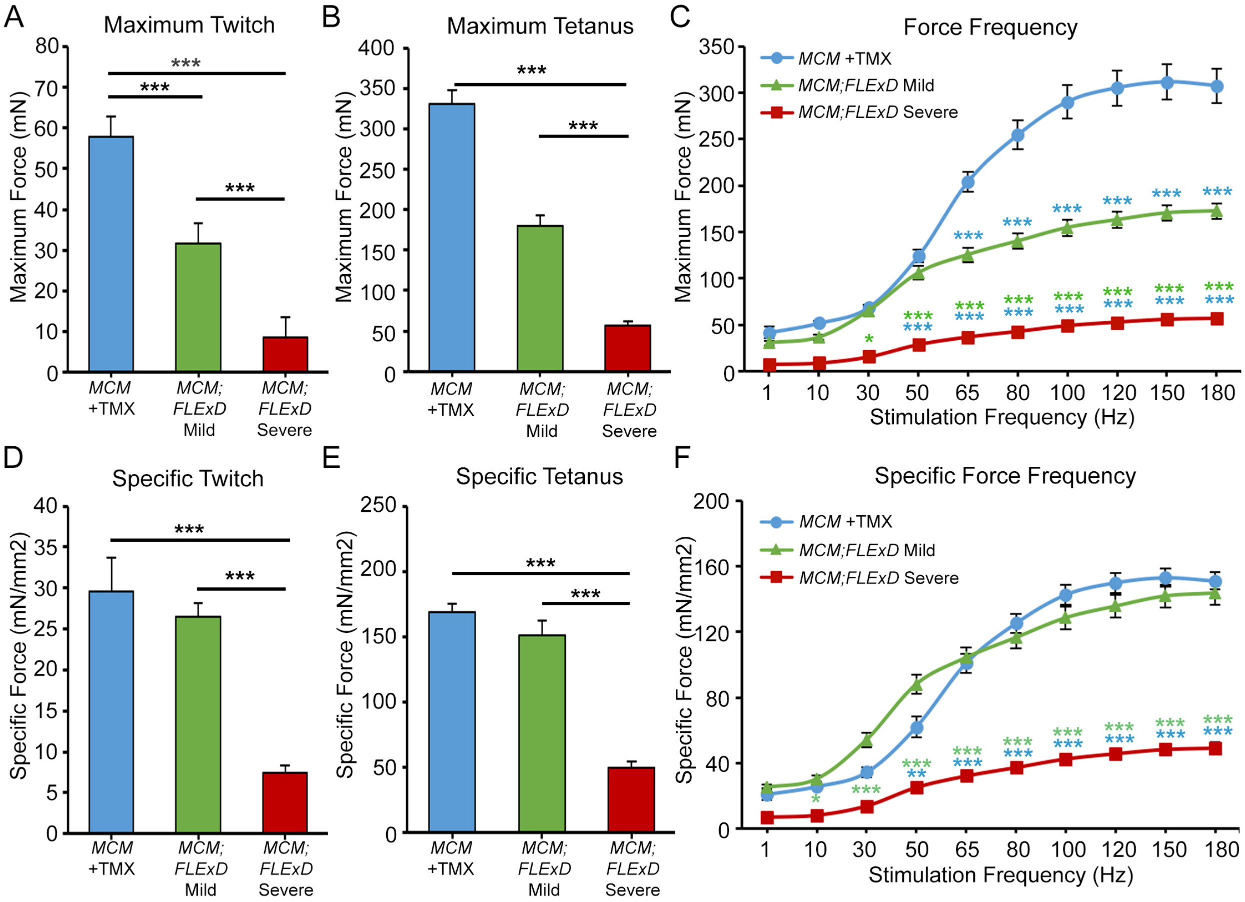

Figure S8: Maximum and specific isometric forces of the male mild and severe FSHD-like mouse models. EDL muscles from *ACTA1-MCM* +TMX (MCM +TMX, control, blue, n=5), mild model (*MCM;FLExD* mild, green, n=9), and severe model (*MCM;FLExD* severe, red, n=11) were assayed at SD10 for A) maximum twitch force, B) maximum tetanus force, C) force frequency, and then normalized for muscle CSA to provide D) specific twitch force, E) specific tetanus force, and F) specific force frequency. Data is mean +/- S.D. Significance = * $p<0.05$, ** $p<0.01$, ***$p<.001$; blue asterisks indicate significance compared with the *ACTA1-MCM* control and green asterisks indicate significance compared with mild model.

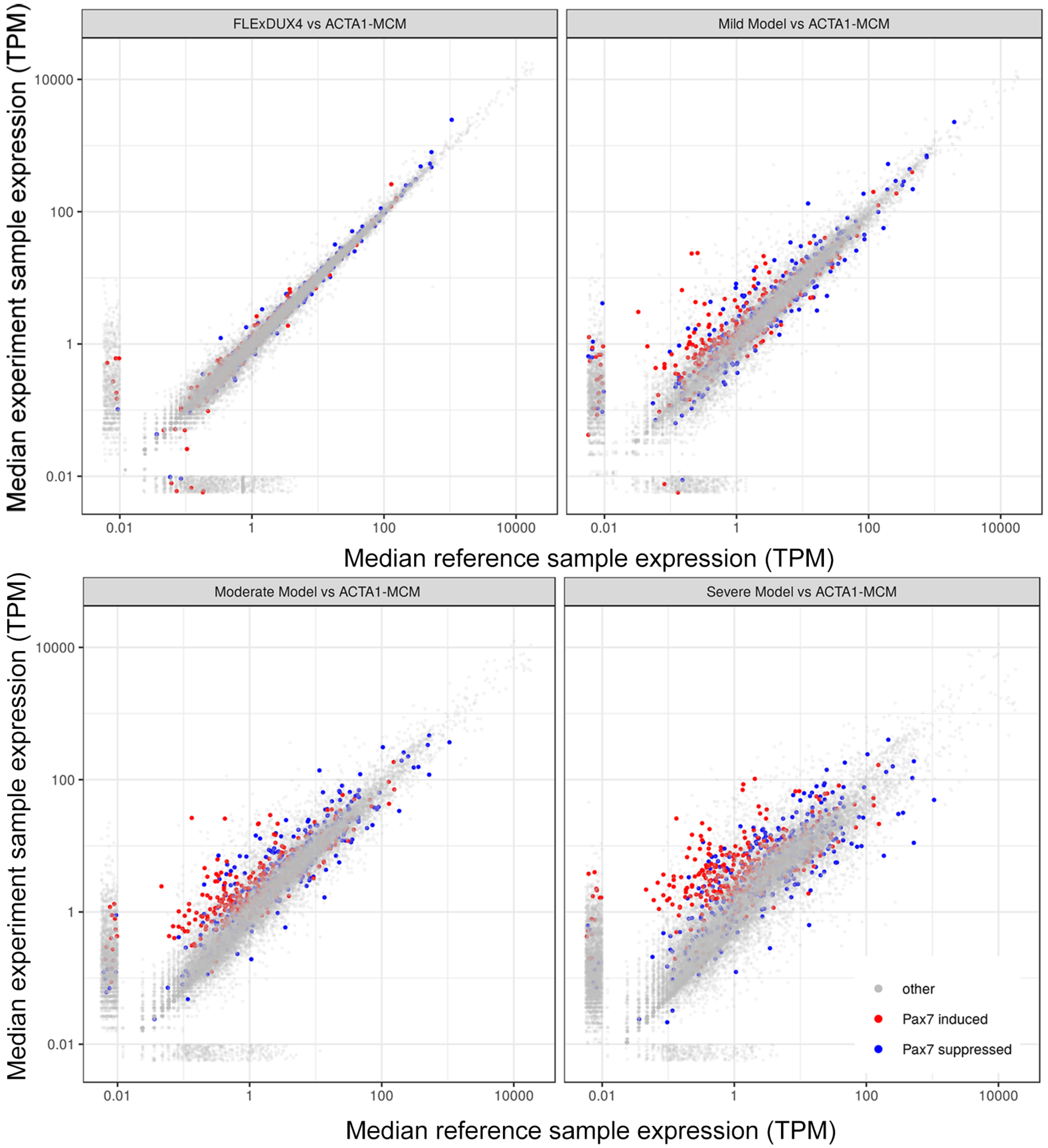

Figure S9: Pax7 target genes are not significantly misexpressed in the FSHD-like mouse models. RNA-seq data was analyzed for expression of Pax7 target genes (Banerji and Zammitt, 2019; biomarker_files.rd) in all three FSHD-like severity models as well as the FLExD/+ alone. Genes known to be induced by Pax7 are in red, genes known to be repressed by Pax7 are in blue. Pax7-induced genes appear slightly up in the moderate and severe models; however, these changes are not significant as a group.

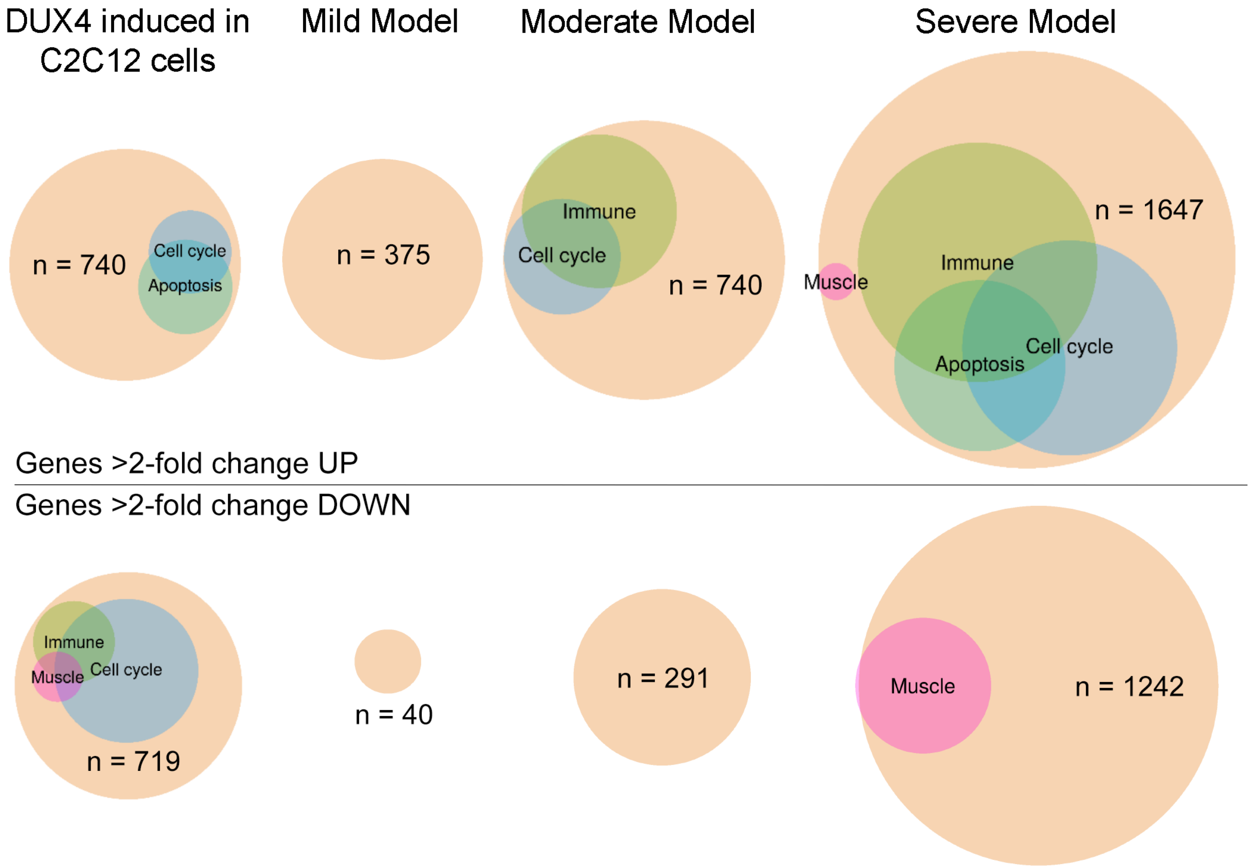

Figure S10: GO enrichment analysis of differentially expressed genes (>2-fold) in the different FSHD-like severity model mice. GO enrichment analysis for DUX4-induced genes identified in (left to right) C2C12 cells [25], mild, moderate, and severe FSHD-like model mice. The size of the beige-colored circles indicates the total number of differentially expressed genes (>2-fold), colored circles indicate the proportion of genes from significantly enriched GO-terms, that are in turn offspring of GO superterms (see methods and Table S4) relating to *mus*. Upregulated genes are represented in the top half and downregulated genes are represented in the lower half. The number of genes in each grouping is indicated (n).

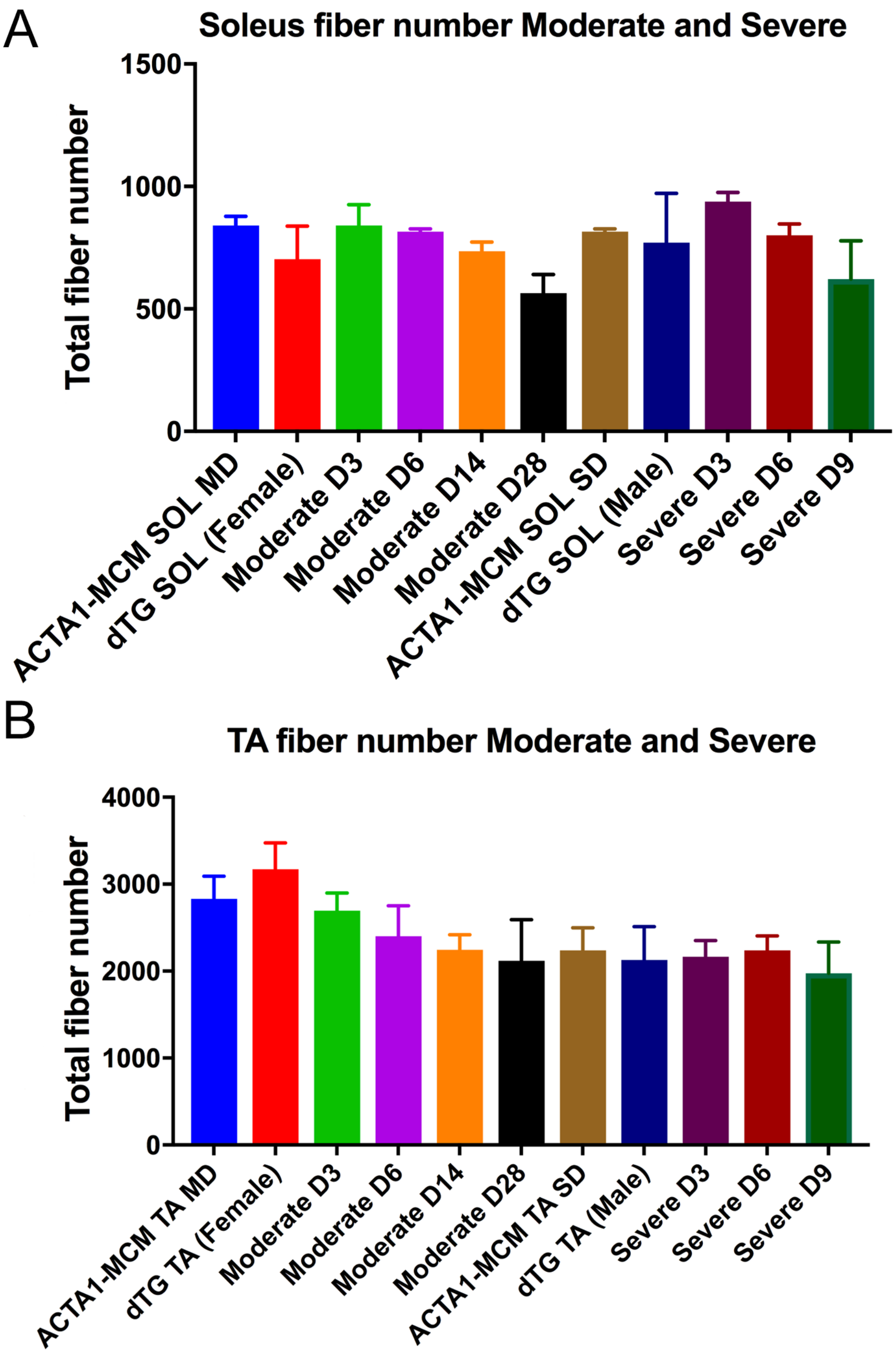

Figure S11: Fiber number per cross-section does not significantly change with severity. Average total myofibers counted during centralized nuclei analysis (Figure 10) for A) soleus and B) tibialis anterior. Although the number of fibers trended down during the course of the pathological progression, the changes did not reach significance ($p<0.05$). Data is mean +/- S.D.

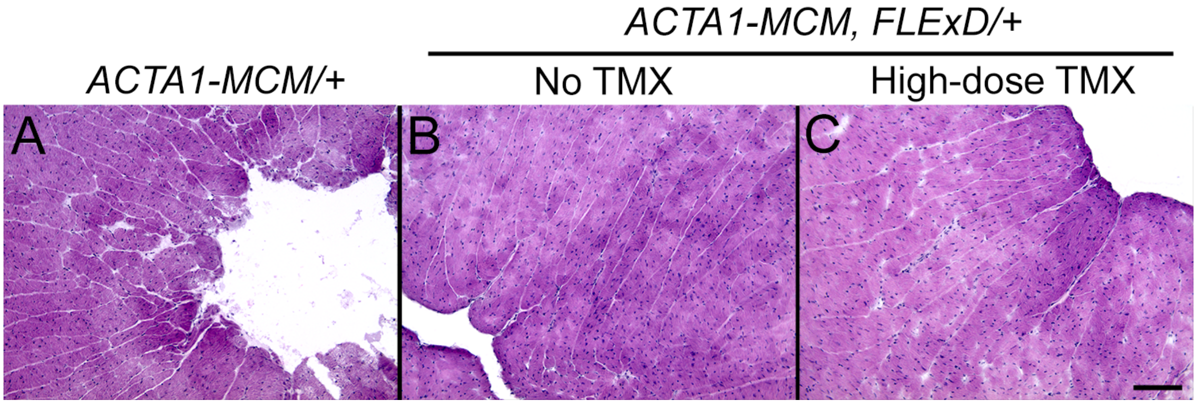

Figure S12: The heart is not affected by TMX treatment in control or bi-transgenic animals. H&E staining of cryosections of heart isolated from A) *ACTA1-MCM* mice, and bi-transgenic *ACTA1-MCM;FLExD* mice B) without TMX (mild model) and C) with the high-dose of TMX (severe model). Scale bar = 100 μm

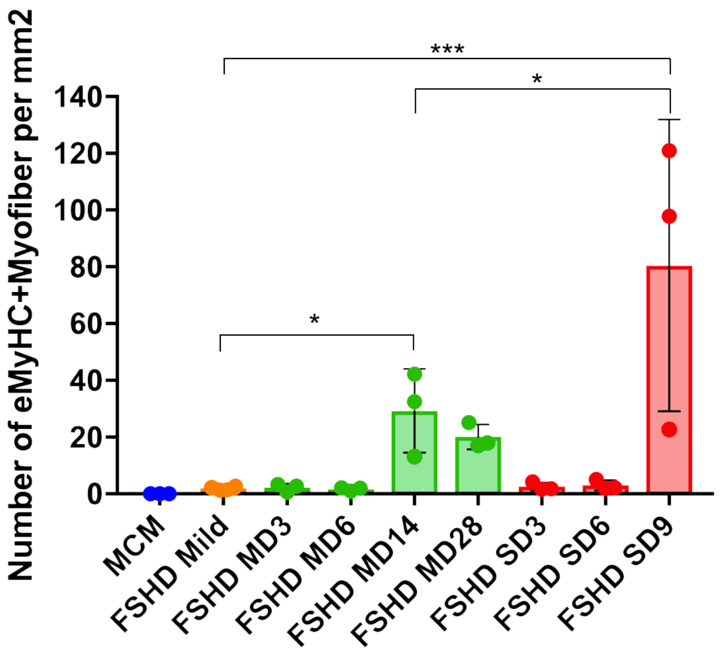

Figure S13: Quantification of eMyHC positive muscle cells in different FSHD-like severity mouse models. Data is mean +/- S.D. Significance is calculated compared to the mild model using one-way ANOVA, uncorrected Fisher's LSD; $p<0.05$ (*), $p<0.001$ (***)

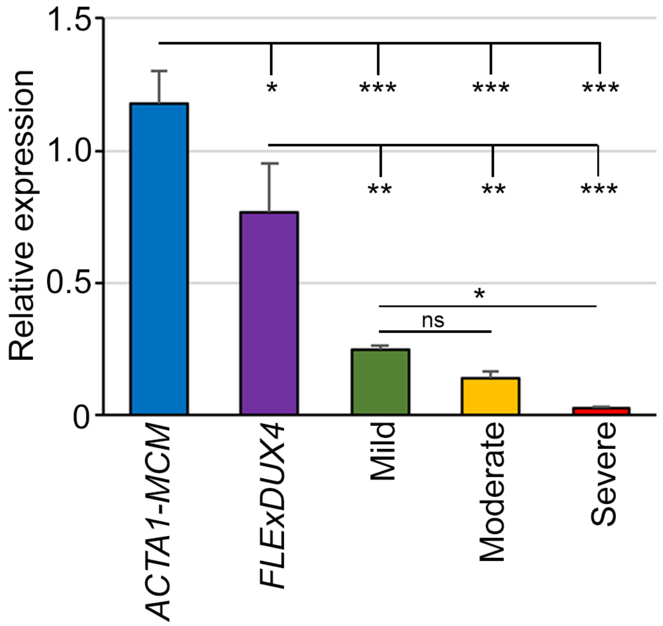

Figure S14: *Mstn* gene expression decreases with increased *DUX4-fl* expression in the FSHD-like mouse models. *Mstn* mRNA levels from 12-13 wk old mice were assayed by qRT-PCR, for the models as indicated. The moderate model was assayed at MD9 and the severe model at SD9. Levels were normalized to *RpL37* mRNA levels. Data is mean +/- S.D. Significance was calculated using one-way ANOVA with uncorrected Fisher's LSD. * $p<0.05$, ** $p<0.01$, *** $p<0.001$, ns= not significant

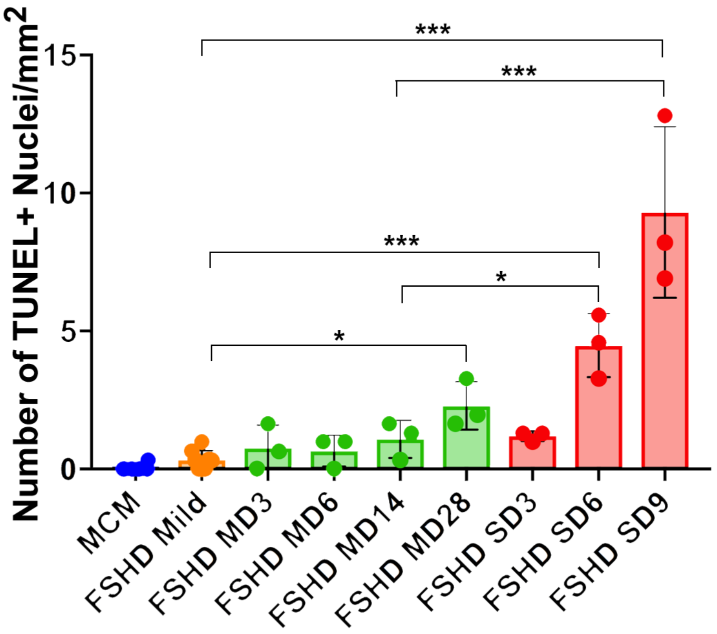

Figure S15: Quantification TUNEL positive nuclei in different FSHD-like severity models. Data is mean +/- S.D. Significance was calculated using one-way ANOVA with uncorrected Fisher's LSD; p<0.05 (*), p<0.01 (**), p<0.001 (***)

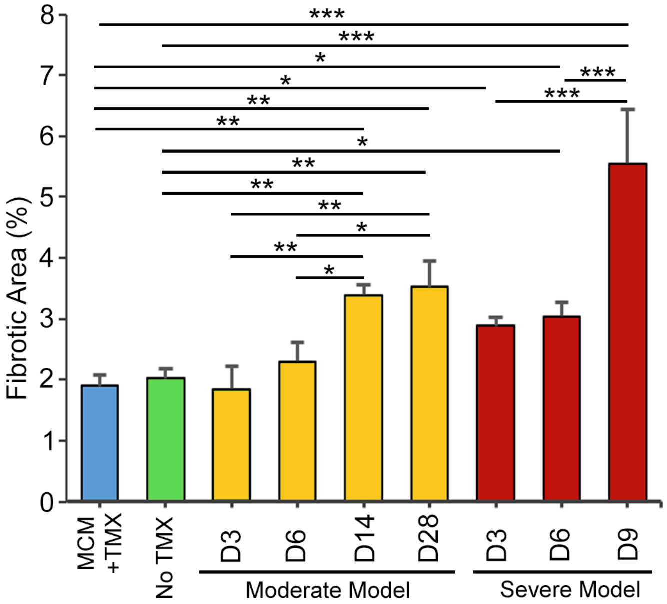

Figure S16: Significant fibrosis in late stages of the moderate and severe FSHD-like mouse models. Summary of Sirius red staining for tibialis anterior muscle sections for mild (green), moderate (yellow), and severe (red) FSHD-like mouse models compared with *ACTA1-MCM* control (blue). Data is plotted as percent fibrotic area. Data is mean +/- S.D. Significance was calculated using the least significant difference test, with *= p<.05, **= p<.01, and ***= p<.001.

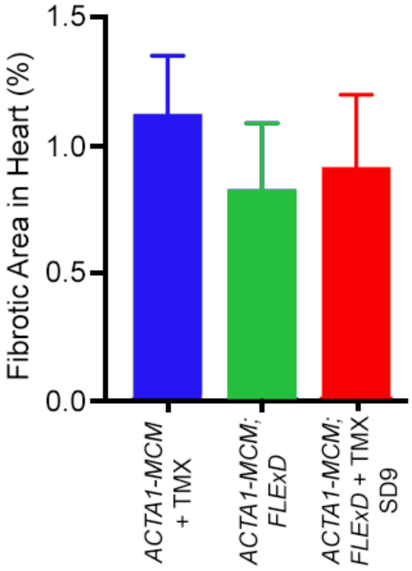

Figure S17: Cardiac muscle from FSHD-like mouse models shows no signs of increased fibrosis. Hearts were isolated from the same mice assayed in Figure 14 for skeletal muscle fibrosis. Control *ACTA1-MCM* mice (blue), bi-transgenic mild model mice (green) and the severe model mice at SD9 (red) show no significant differences in heart fibrosis. Data is mean +/- S.D.
